# Assessment of Different Irrigation Thresholds to Optimize the Water Use Efficiency and Yield of Potato (*Solanum tuberosum* L.) Under Field Conditions

**DOI:** 10.3390/plants14111734

**Published:** 2025-06-05

**Authors:** Rodrigo Mora-Sanhueza, Ricardo Tighe-Neira, Rafael López-Olivari, Claudio Inostroza-Blancheteau

**Affiliations:** 1 Programa de Doctorado en Ciencias Agropecuarias, Facultad de Recursos Naturales, Universidad Católica de Temuco, P.O. Box 15-D, Temuco 4780000, Chile; rmora2023@alu.uct.cl; 2 Laboratorio de Fisiología y Biotecnología Vegetal, Departamento de Ciencias Agropecuarias y Acuícolas, Facultad de Recursos Naturales, Universidad Católica de Temuco, P.O. Box 15-D, Temuco 4780000, Chile; 3 Núcleo de Investigación en Producción Alimentaria, Facultad de Recursos Naturales, Universidad Católica de Temuco, P.O. Box 15-D, Temuco 4780000, Chile; 4 Instituto de Investigaciones Agropecuarias, INIA Carillanca, km 10 camino Cajón-Vilcún s/n, Casilla 929, Vilcún 4880000, Chile

**Keywords:** potato, deficit irrigation, WUE_int_, photosynthesis, chlorophyll fluorescence

## Abstract

The potato (*Solanum tuberosum* L.) is highly dependent on water availability, with physiological sensitivity varying throughout its phenological cycle. In the context of increasing water scarcity and greater climate variability, identifying critical periods where water stress negatively impacts productivity and tuber quality is essential. This study evaluated the physiological response of potatoes under different deficit irrigation strategies in field conditions, and aimed to determine the irrigation reduction thresholds that optimize water use efficiency without significantly compromising yield. Five irrigation regimes were applied: well-watered (T1; irrigation was applied when the volumetric soil moisture content was close to 35% of total water available), 130% of T1 (T2, 30% more than T1), 75% of T1 (T3), 50% of T1 (T4), and 30% of T1 (T5). Key physiological parameters were monitored, including gas exchange (net photosynthesis, stomatal conductance, and transpiration), chlorophyll fluorescence (Fv’/Fm’, ΦPSII, electron transport rate), and photosynthetic pigment content, at three critical phenological phases: tuberization, flowering, and fruit set. The results indicate that water stress during tuberization and flowering significantly reduced photosynthetic efficiency, with decreases in stomatal conductance (gs), effective quantum efficiency of PSII (ΦPSII), and electron transport rate (ETR). In contrast, moderate irrigation reduction (75%) lowered the seasonal application of water by ~25% (≈80 mm ha^−1^) while maintaining commercial yield and tuber quality comparable to the fully irrigated control. Intrinsic water use efficiency increased by 18 ± 4% under this regime. These findings highlight the importance of irrigation management based on crop phenology, prioritizing water supply during the stages of higher physiological sensitivity and allowing irrigation reductions in less critical phases. In a scenario of increasing water limitations, this strategy enhances water use efficiency while ensuring the production of tubers with optimal commercial quality, promoting more sustainable agricultural management practices.

## 1. Introduction

The potato (*Solanum tuberosum* L.) ranks third among food crops worldwide, with an annual output close to 376 Mt harvested from about 17.5 million ha [1]. In Chile, it is the most widespread horticultural crop, covering ≈40.800 ha and yielding ≈1.146 Mt in the 2023/2024 season [2]. Globally, about 52% of the potato area is managed by resource-poor farmers in developing countries, which are subject to increasing water scarcity [3,4]. 

Potato yield is strongly influenced by water availability, as it is very sensitive to water deficits due to its shallow root system, which restricts its ability to extract water from deeper soil layers [5,6,7]. Potato irrigation requirements vary widely according to climate and soil type: in the United Kingdom they are estimated to be between 143 and 313 mm per season [8], while in semi-arid regions of the southern United States they can exceed 600 mm [9]. In La Araucanía, Chile, annual precipitation (1.200–1.500 mm) is considerably higher; however, about 70% is concentrated between April and September, so boreal summers are characterized by a marked water deficit (ET₀ > 6 mm day^−1^), making supplementary irrigation necessary [10,11]. This mismatch between supply and demand means it is essential to optimize water use during critical periods of crop development.

The impact of water deficit on potatoes varies throughout their phenological cycle, affecting multiple physiological processes that are key determinants of tuber yield and commercial quality. The most affected physiological variables include stomatal conductance [gs (mol H_2_O m^−2^ s^−1^)], net photosynthesis [Pn (μmol CO_2_ m^−2^ s^−1^)], transpiration rate [E (mol H_2_O m^−2^ s^−1^)], intrinsic water use efficiency (WUE_int_), chlorophyll fluorescence (Fv’/Fm’, ΦPSII, ETR), and photosynthetic pigment content [12,13,14]. Water stress-induced stomatal closure restricts CO_2_ intake, reducing net photosynthetic rate (Pn) and, consequently, biomass accumulation and the translocation of photoassimilates to tubers [15,16]. Structurally, water deficits can affect canopy morphology, reducing the leaf area index (LAI), stolon number and size, and root development, which ultimately compromise tuber formation and filling [6,17,18,19].

Several studies have identified tuberization and flowering as the most water deficit-sensitive periods, with significant impacts on photosynthetic efficiency and final yield [13,17,20]. In contrast, during emergence and senescence, potatoes exhibit greater tolerance to water stress, allowing the implementation of deficit irrigation strategies without substantially affecting productivity [21]. These findings have driven the development of controlled deficit irrigation approaches such as sustained deficit irrigation (SDI) and partial root zone drying (PRD), which aim to optimize water efficiency by reducing irrigation in less demanding stages and prioritizing water supply during critical phenological phases [22]. 

In this sense, this study hypothesizes that an irrigation reduction threshold can be used to maximize water use efficiency (WUE_int_) without compromising potato production. Therefore, the aim of this work was to identify the reduction thresholds that maximize WUE_int_ without materially sacrificing commercial tuber yield of *S. tuberosum* under field conditions.

## 2. Materials and Methods

### 2.1. Study Area

All experiments were conducted at the Carillanca Regional Research Center of the Institute of Agricultural Research (INIA), La Araucanía Region, Chile (38°41′ S, 72°24′ W, 188 m.a.s.l.). 

A drip-irrigated Puyehue-INIA variety [23], a Chilean potato variety (*Solanum tuberosum* L.), was grown in a total experimental area of 282 m^2^ in a flat field (56.2 m^2^ per irrigation strategy) during two growing seasons: 2021/2022 and 2022/2023. The planting density was 0.25 m between plants × 0.75 m between rows, with planting beginning in mid-November (DOY (Day of year) 314 or November 17) for the first season and DOY 341 (6 December) for the second season. The effective rooting depth (Pe effective) was 30 cm (with more than 80% active roots) under well-watered conditions, determined using a soil pit dug at the end of each season.

The experimental site has a temperate climate [10], and the soil is classified as Temuco series (Andisol, Typic Hapludands family), with a silty loam texture [24]. The soil characteristics included 13.4% organic matter content, 0.79 g cm^−3^ bulk density, 0.52 m^3^ field capacity, and 0.27 m^3^ wilting point. Fertilization was applied at a total rate of 220 kg P ha^−1^ (at planting), 118 kg K ha^−1^, and 240 kg N ha^−1^ (50% at planting and 40% before potato flowering). Pest and disease management were carried out preventively in both seasons through the application of a broad-spectrum insecticide (pyrethroid + neonicotinoid) and a fungicide (carbamates + pyridinylmethyl–benzamide) specific to potato crops, following product recommendations. Weed control involved pre-emergence herbicide application (metribuzin) and manual weeding every 10–15 days throughout the season.

In the 2021–2022 and 2022–2023 seasons, evaluations were conducted at key phenological stages according to the BBCH (Biologische Bundesanstalt, Bundessortenamt and Chemical Industry) scale [25]. For the 2021–2022 season, the key dates are as follows: tuberization on 23 December 2021 (DOY 357; BBCH 45), peak flowering on January 17, 2022 (DOY 17; BBCH 69), and fruit formation onset on 31 January 2022 (DOY 31; BBCH 75). For the 2022–2023 season, evaluations were carried out at the same phenological stages but on different dates: tuberization on 20 January 2023 (DOY 20; BBCH 45), peak flowering on 10 February 2023 (DOY 41; BBCH 69), and fruit formation onset on 21 February 2023 (DOY 52; BBCH 75).

### 2.2. Irrigation Water Treatments and Water Management

The potato plants in this study were subjected to five different irrigation strategies: well-watered (T1), over-irrigation by applying 30% more than T1 (T2), application of 75% of T1 (T3), application of 50% of T1 (T4), and application of 30% of T1 (T5). The amount of water applied for each irrigation strategy (subplot of 18.75 m^2^) was defined using the drip flow rate per plant (Netafim Ltd., Tel Aviv, Israel) by manually inserting a pressure-compensating button dripper into the drip irrigation lines (one per plant). Discharges of 4.0 (T1), 3.0 (T3), 2.0 (T4), and 1.2 L h^−1^ (T5) were used. All drippers were spaced 0.25 m apart. In the case of over-watered T2 treatments, two pressure-compensating button drippers per plant with a discharge of 4.0 and 1.2 L h^−1^ each (total 5.2 L h^−1^), spaced at 0.25 m, were used. The irrigation scheduling for T1 was calculated based on the concept of total available soil water (TAW; mm), soil water depletion fraction (p), and readily available soil water (RAW; mm) [26]. A “p” equal to 0.35 [25] was used, and this factor was maintained throughout the growing season [27,28,29]. Irrigation events were carried out when 35% of the RAW of the effective root zone was depleted. The volumetric soil moisture for each irrigation level was monitored using frequency domain reflectometry (FDR; ECH2O GS-1 and GS-3, METER Group, Inc., Pullman, WA, USA). The frequency of irrigation was defined using the FDR sensor reading from the well-watered condition (T1). The irrigation time was determined by incorporating the concept of RAW, the discharge of the drippers, and the efficiency of irrigation. The effective daily precipitation (Pef) was determined according to Ref [27] These values were incorporated as a water contribution for irrigation scheduling [26].

### 2.3. Soil Moisture

After hilling of the potato, five FDR probes were installed in a representative area for continuous measurements of soil volumetric moisture throughout the growing season for each irrigation level evaluated. For each irrigation level, ECH2O GS-3 FDR probes were installed at a depth of 30 cm. Each set of FDR probes was used to measure soil moisture variation in the effective rooting zone. All readings were recorded at 15-min intervals using three different data loggers (Em50 solar data logger, METER Group, Inc., Pullman, WA, USA). Before installing the sensors in the soil, they were externally calibrated in an undisturbed soil cube removed from the experimental site following the gravimetric method according to Ref. [30].

### 2.4. Chlorophyll a Fluorescence Parameter of PSII

Chlorophyll fluorescence was measured in light adaptation conditions, where the maximum quantum yield [Fv’/Fm’ = (Fm’ − F0’)/Fm’] was calculated according to Ref. [31], and the effective quantum yield of PSII [ΦPSII = (Fm’ − Fs)/Fm’] and the electron transport rate (ETR = ΦPSII × α × β × PPFD) were calculated according to Ref. [32]. *In vivo* measurements were performed using a portable infrared CO_2_ analyzer equipped with a Li-Cor LR6400 measuring cuvette with its light source and fluorescence chamber (LCF 6400-40; LI-COR Inc., Lincoln, NE, USA) during the light period (9:00 to 12:00 h).

### 2.5. Gas Exchange Measurements

Gas exchange measurements were conducted using an IRGA (LI-6400xt, LI−COR Inc., Lincoln, NE, USA), considering a flow rate of 300 μmol, photosynthetically active radiation (PAR) of 1000 μmol photons m^−2^ s^−1^ (saturation point), at 20 °C, 400 μmol CO_2_ mol^−1^, and 55–60% relative humidity. Measurements were performed using the second leaflet of the third fully expanded leaf after two hours of light adaptation. Photosynthesis (Pn) (µmol CO_2_ m^−2^s^−1^), stomatal conductance [gs (mmol H_2_O m^−2^s^−1^)], and transpiration (E) (mol H_2_O m^−2^s^−1^) were also determined. Water use efficiency (WUE_int_) was estimated by means of the quotient between Pn and gs [33].

### 2.6. Quantification of Photosynthetic Pigments

Photosynthetic pigments chlorophyll *a* (Chl *a*), chlorophyll *b* (Chl *b*), and total carotenoids (Car) were determined according to the method described by Ref. [34] with modifications to enhance extraction efficiency and analytical accuracy. Frozen leaf tissue (0.10 g) was ground in liquid N_2_ with a trace of CaCO₃ and extracted in 1.0 mL 96% ethanol. After centrifugation (13.000× *g*, 5 min, 4 °C), the pellet was re-extracted with 1.0 mL methanol; both supernatants were pooled and adjusted to 2.0 mL (dilution factor = 2). For spectrophotometry, 250 µL extract was mixed with 750 µL ethanol. All steps were performed in duplicate. The pigments were measured using a spectrophotometer (Biobase, BK-UV1800, Shandong, Jinan, China) at absorbances of 665 nm (Chl *a*), 649 nm (Chl *b*), and 470 nm (carotenoids). Finally, the pigments were quantified according to the formulas: chlorophyll *a* = 15.65A_665_ − 7.34A_653_, chlorophyll *b* = 27.05A_649_ − 11.21A_665_, and carotenoids = (1000A_470_ − 2.86 × Ca − 129.2 × Cb)/245 and expressed in (mg g^−1^ FW). All pigment values were first obtained on a fresh weight basis (mg g^−1^ FW) and then converted to dry weight (mg g^−1^ DW) using the measured leaf water content (84.7 ± 1.9%, n = 18) to ensure unit consistency throughout this study.

### 2.7. Yield Estimation

To determine the yield, the measured variables were total number of tubers (TNT), number of commercial tubers (CNT), total tuber weight (TTW), and commercial tuber weight (CTW), including tubers with a diameter of ≥2 cm. Yield per hectare was obtained using the equation of Ref. [35], which considers three basic components of yield: yield (kg ha^−1^) = 1875 plants × number of tubers per plant × average weight of fresh tuber (kg). Finally, the yield was expressed in tons per hectare (t ha^−1^).

### 2.8. Experimental Design and Statistical Analysis

A randomized complete block design (RCBD) with three biological replicates (i.e., independent plants per treatment) and three technical replicates (i.e., repeated measurements per plant) was used to evaluate the physiological responses of *Solanum tuberosum* under different irrigation levels across three phenological stages during two growing seasons. A total of 2000 potato plants were used, distributed in four plots per treatment, with 100 plants per plot, ensuring adequate representation and statistical power for each irrigation regime.

The spatial arrangement of blocks and the allocation of treatments are illustrated in Appendix A Figure A4. Gas exchange, chlorophyll fluorescence parameters, and photosynthetic pigments were analyzed relative to the corresponding control for each measurement day.

Prior to statistical analyses, the dataset was evaluated for normality (Shapiro–Wilk test) and homoscedasticity (Levene’s test). Residual plots and quantile–quantile (QQ) plots were inspected to validate the assumptions required for parametric analyses.

To explore relationships between physiological and biochemical parameters, a Pearson correlation and principal component analysis (PCA) was performed with Z-score normalization, enabling the identification of the most influential variables that respond to the irrigation regimes. A Pearson correlation matrix was computed to assess relationships between gas exchange, photochemical efficiency, and pigment concentration, and visualized using a heatmap.

Two-way ANOVA was conducted to evaluate the effects of irrigation treatments and phenological stages on gas exchange, fluorescence parameters, and chlorophyll content, followed by Tukey’s HSD test for post-hoc comparisons. In addition to classical significance testing using *p*-values, Bayes factor (BF₁₀) values were calculated using the BayesFactor package (prior JZS, r = 0.707). The BF quantifies the relative evidence for H₁ over H₀ in probabilistic units, overcoming the arbitrariness of the α threshold and providing a direct estimate of both effect size and strength of evidence. The interpretation scale used was anecdotal (BF₁₀ < 3), moderate (3–10), strong (10–30), very strong (30–100), and decisive (>100).

To classify treatments based on physiological traits, a hierarchical clustering analysis (HCA) was performed using Euclidean distance and Ward’s method, enabling the identification of treatment groups with similar physiological responses. A multiple linear regression (MLR) model was applied to examine the relationship between net photosynthesis (Pn) and physiological variables [stomatal conductance (gs), transpiration (E), and effective quantum yield of PSII (ΦPSII)]. The assumptions of the model linearity, normality of residuals, homoscedasticity, and multicollinearity were evaluated to ensure statistical robustness. The model was structured as follows:

Pn = β_0_ + β_1_ × gs + β_2_ × E + β_3_ × ΦPSII

where:

- Pn is the net photosynthesis rate (μmol CO_2_ m^−2^ s^−1^);
- gs is stomatal conductance (mol H_2_O m^−2^ s^−1^);
- E is transpiration rate (mmol H_2_O m^−2^ s^−1^);
- ΦPSII is the effective quantum yield of PSII;
- β₀ represents the intercept;
- β₁, β_2_, and β₃ are the regression coefficients estimating the contribution of each predictor to Pn.

All statistical analyses were performed in RStudio (v.22.0.3) using the following packages: ggplot2, dplyr, tidyr, ggfortify, corrplot, factoextra, cluster, car, lmtest, and BayesFactor, with a significance level of α = 0.05.

## 3. Results

### 3.1. Meteorological Measurements and Soil Moisture Conditions

**Figure A1:**
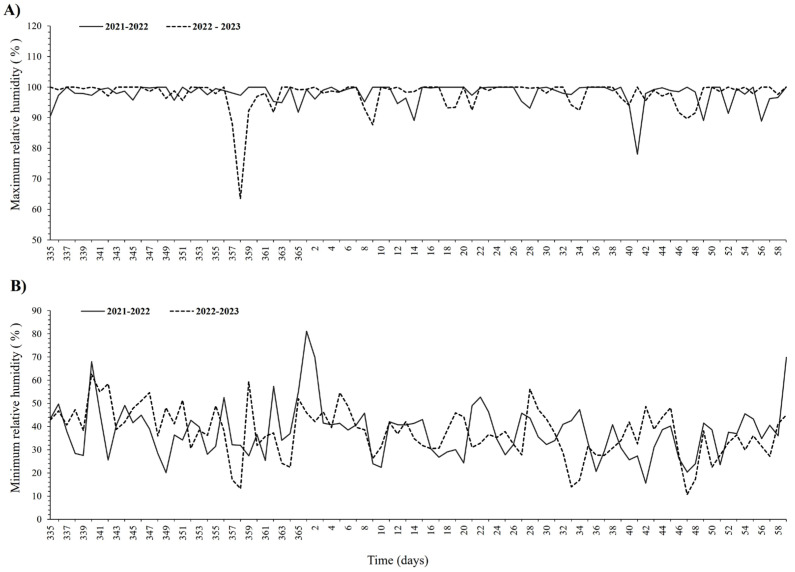
Daily variation in relative humidity recorded by an automatic weather station (EMA) located near the study site (latitude: −38.68, longitude: −72.41, elevation: 200 m.a.s.l.) during the 2021–2022 and 2022–2023 growing seasons. Panel (**A**) shows daily maximum relative humidity (%), while panel (**B**) shows daily minimum relative humidity (%) over the monitored period.

**Figure A2:**
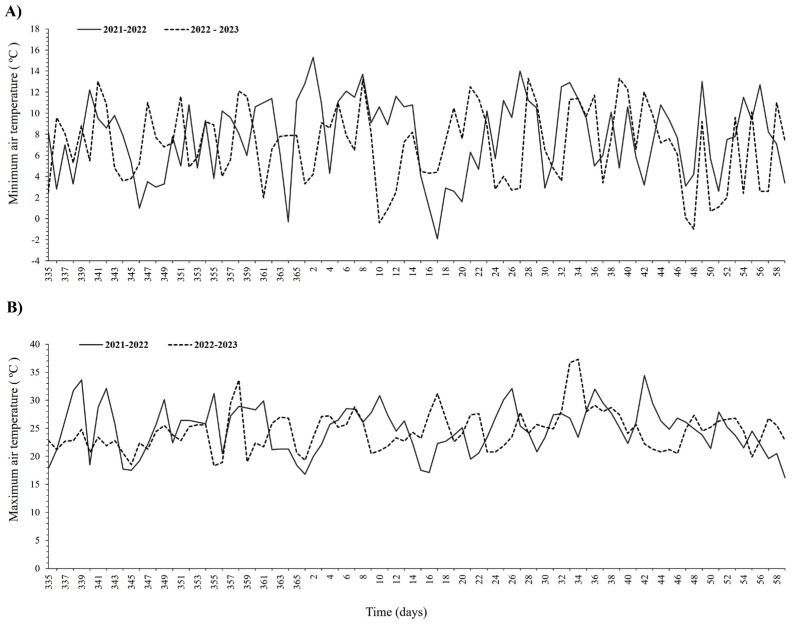
Daily variation in air temperature recorded by an automatic weather station (EMA) located near the study site (latitude: −38.68, longitude: −72.41, elevation: 200 m.a.s.l.) during the 2021–2022 and 2022–2023 growing seasons. Panel (**A**) shows daily minimum air temperature (°C), and panel (**B**) shows daily maximum air temperature (°C) over the monitored period.

**Figure A3:**
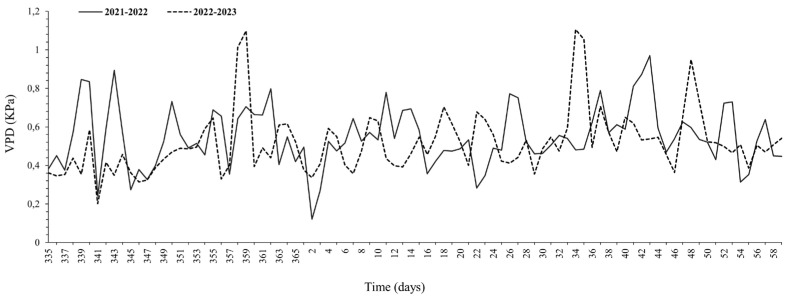
Daily variation in vapor pressure deficit (VPD) estimated using maximum and minimum air temperature and relative humidity, recorded by an automatic weather station near the study site during the 2021–2022 and 2022–2023 seasons.

**Figure A4:**
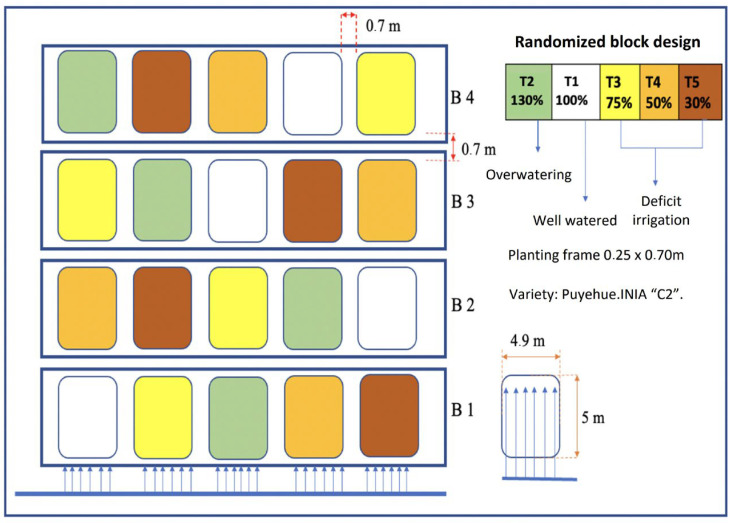
Plot design in experiment.

Seasonal climate differed between growth seasons (Appendix A Figure A1, Figure A2 and Figure A3). Total cumulative precipitation (TCP) from planting to 15 d before harvest reached 180 mm in 2021/2022 and 85 mm in 2022/2023 (↓53%). The second season nevertheless showed a 73% surge in early vegetative rainfall (DOY 344–362), followed by a pronounced summer deficit. Reference evapotranspiration (ET₀) peaked at 6.3 mm d^−1^ (DOY 350, stem elongation) and 7.8 mm d^−1^ (DOY 46, peak flowering). Air temperature spanned 1–34 °C (mean daily max 23.5 ± 2 °C), while minimum relative humidity occasionally dropped below 25%. The resulting vapor pressure deficit (VPD) fluctuated between 0.2 and 1.2 kPa (mean 0.55 ± 0.25 kPa), imposing moderate atmospheric demand. Detailed trends in relative humidity, temperature, and VPD are presented in Appendix A, Figure A1, Figure A2 and Figure A3.

**Figure 1:**
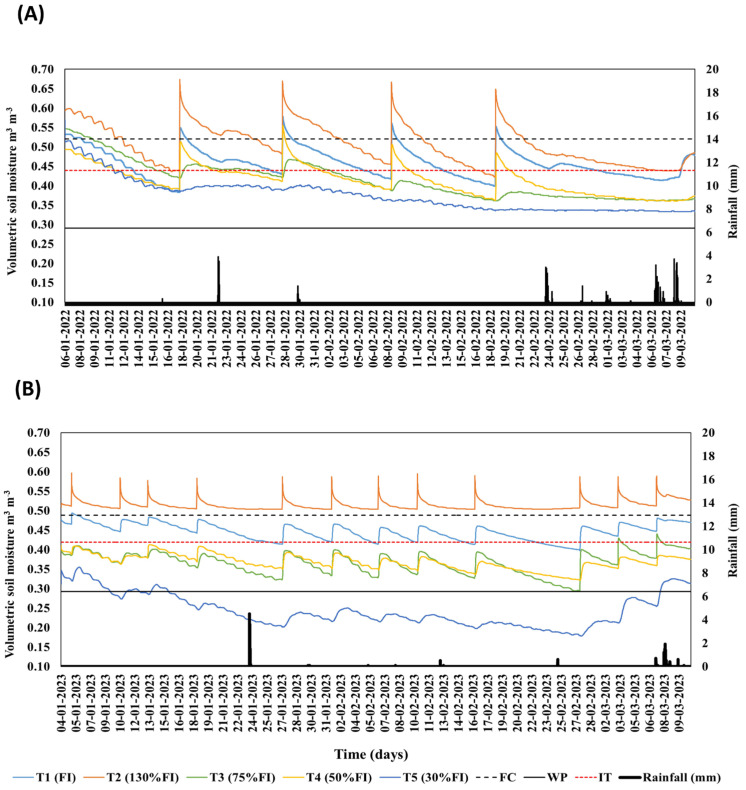
Temporal dynamics of soil volumetric water content (m^3^ m^−3^) and daily rainfall (R, mm) during the 2021/2022 (**A**) and 2022/2023 (**B**) seasons. Irrigation treatments (T) were T1 = 100 % of crop evapotranspiration replacement (full irrigation), T2 = 130 %, T3 = 75 %, T4 = 50 %, and T5 = 30 % of T1. The red dashed line marks the irrigation threshold (IT), while the two black lines indicate soil water content at field capacity (FC, dashed line) and permanent wilting point (WP, solid line), respectively. Vertical bars represent measured precipitation.

Soil water monitoring (GS-3 probes at 20 and 30 cm) confirmed that the well-watered reference (T1) never exceeded the 35% readily available water depletion threshold. Across treatments, seven irrigation events (2021/2022) and eight (2022/2023) plus ≤3 effective rainfall events per season maintained volumetric water content within the prescribed target boundaries (Figure 1, Table 1). Cumulative irrigation + effective rainfall for T1 was 315 mm (2021/2022) and 323 mm (2022/2023), providing the baseline against which the four deficit/over-irrigation regimes were imposed.

### 3.2. Photosynthetic Performance in S. tuberosum Subjected to Deficit Irrigation Strategies

**Figure 2:**
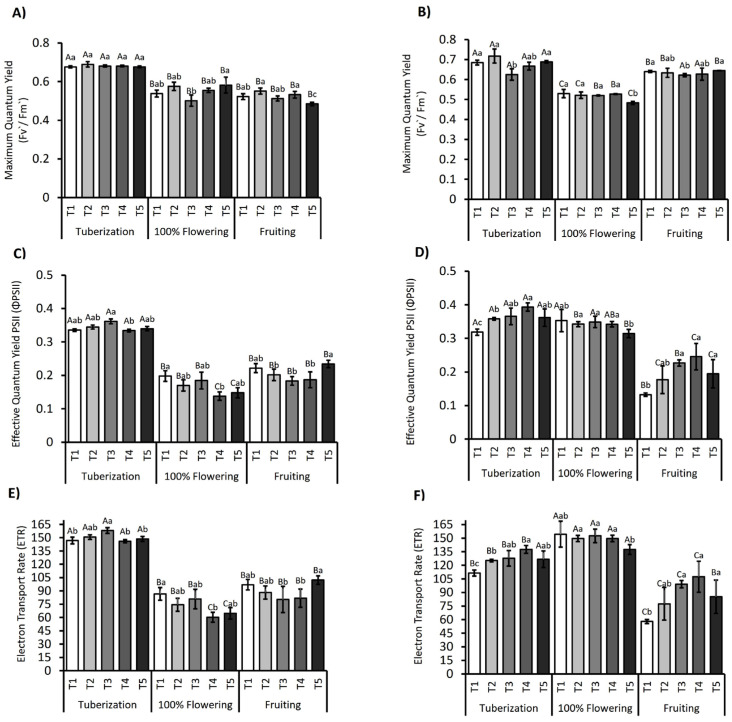
Maximum photochemical efficiency (Fv’/Fm’), effective quantum yield of PSII (ΦPSII), and electron transport rate (ETR) in the *S. tuberosum* var. Puyehue-INIA during the 2021/2022 (**A**,**C**,**E**) and 2022/2023 (**B**,**D**,**F**) growing seasons. Plants were subjected to five irrigation treatments: well-watered (T1, 100%), over-irrigated (T2, 130%), and deficit irrigation conditions (T3, 75%; T4, 50%; T5, 30%). Measurements were taken at three phenological stages: tuberization, peak flowering, and fruiting. Bars represent the mean ± standard error (SE). Different capital letters indicate significant differences between treatments across phenological stages, while lowercase letters indicate significant differences within each phenological stage, according to Tukey’s HSD test (*p* ≤ 0.05).

In Figure 2, the photosynthetic performance under deficit irrigation in *S. tuberosum* is shown. During the tuberization stage, the Fv’/Fm’ showed minimal variation between irrigation treatments, remaining around 0.7 in both seasons. In contrast, during the peak flowering and fruiting stages, a significant reduction in Fv’/Fm’ was observed in all treatments (*p* ≤ 0.05) for both seasons (Figure 2A,B). ANOVA analysis indicated that the “phenological stage” factor had a significant impact on Fv’/Fm’ (*p* < 0.001), while the “treatment” and TxS interaction factors were not statistically significant (*p* > 0.05). During the fruiting stage in the 2021/2022 season, the T5 treatment showed recovery in Fv’/Fm’. In the second season, both the phenological stage and the treatments and their interaction significantly influenced Fv’/Fm’ (*p* < 0.001), indicating that the Fv’/Fm’ response to irrigation treatments depends on the phenological stage. Further analysis focuses on the implications of these results, considering factors such as climatic variability and agronomic management practices that could have influenced the performance of the treatments.

The ΦPSII showed similar behavior, with significant variations depending on the phenological stage (*p* < 0.001), as well as on the effect of the treatment and its interaction (TxS) (*p* < 0.05). During tuberization, ΦPSII remained relatively stable for the 2021/2022 season (Figure 2C), while in the same season, a reduction in ΦPSII was observed during flowering, accentuated in the T4 and T5 treatments. However, in fruiting, a recovery of T5 was recorded. A similar behavior was observed for the 2022/2023 season (Figure 2D); during flowering, a general decrease in ΦPSII was observed for all treatments, which was more pronounced in T5. During fruiting, a decrease was recorded in all regimes compared to flowering. However, in the T3 and T4 treatments, an increase of 1.8- and 1.5-fold was observed compared to the T1 control.

Similarly, ETR showed significant differences between treatments and the interaction between factors and the phenological stage (*p* < 0.001). During tuberization in the 2021/2022 season, the T3 treatment presented the highest values, as did the T3 and T4 treatments in the 2022/2023 season (Figure 2E,F), with an average of 158.08 and 127.84 µmol e^−1^ m^−2^ s^−1^. During the flowering stage of the 2021/2022 season, a reduction was observed in all regimes, accentuated in the T4 and T5 treatments by 58% and 56%, respectively. Meanwhile, in the 2022/2023 season (Figure 2F), an increase in ETR was observed in all treatments during flowering, where, as in the previous season, T5 recorded the lowest values. Finally, during the fruiting stage, a partial recovery of T5 was observed for the 2021/2022 season, while for the 2022/2023 season, a reduction in all treatments compared to flowering was recorded. However, ETR in the T3 and T4 treatments increased 1.7- and 1.8-fold compared to the control.

### 3.3. Gas Exchange Measurement

**Figure 3:**
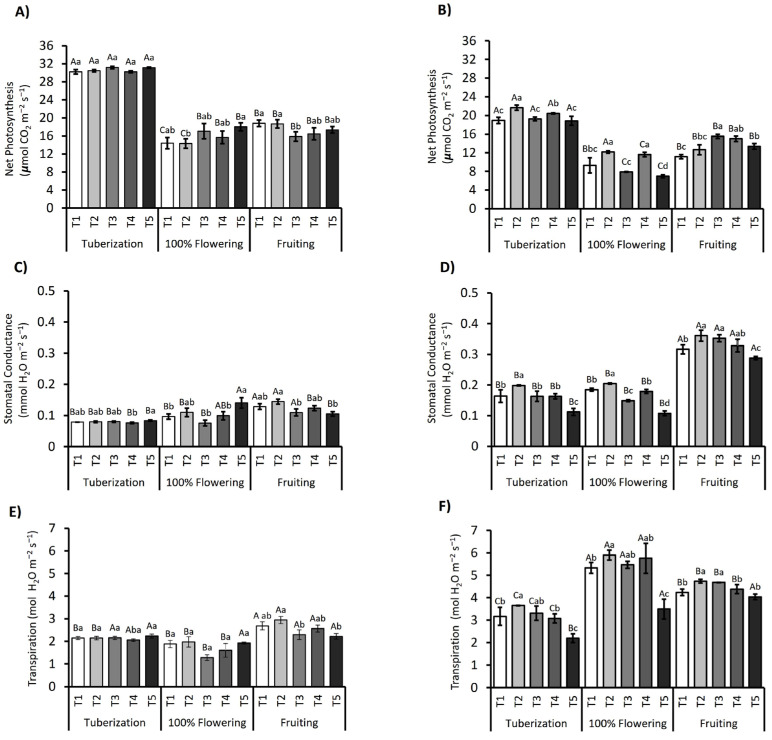
Dynamics of net photosynthesis (Pn), stomatal conductance (gs), and transpiration rate (E) in the *S. tuberosum* var. Puyehue-INIA during the 2021/2022 (**A**,**C**,**E**) and 2022/2023 (**B**,**D**,**F**) growing seasons. Plants were subjected to five irrigation regimes: well-watered (T1, 100%), over-irrigated (T2, 130%), and deficit irrigation treatments (T3, 75%; T4, 50%; and T5, 30%). Measurements were taken at three phenological stages: tuberization, peak flowering, and fruiting. Bars represent the mean ± standard error (SE). Different capital letters indicate statistically significant differences between treatments across phenological stages, whereas lowercase letters indicate differences within each stage, according to Tukey’s HSD test (*p* ≤ 0.05).

Net photosynthesis (Pn) varied significantly throughout the different phenological stages and irrigation treatments, as observed in Figure 3A,B. During the first growing season (2021/2022), statistically significant differences (*p* ≤ 0.05) in Pn were identified in the phenological stage, with the effect of the phenological stage accounting for approximately 85.6% of the total variability observed. For the 2021/2022 season, a reduction in Pn was noted during the flowering stage compared to tuberization. However, treatments T3 and T5 had a higher Pn compared to T1, reaching values of 16 and 17 µmol CO_2_ m^−2^ s^−1^. For the 2022/2023 season, during tuberization, the T2 treatment showed an increase in Pn to 21.64 µmol CO_2_ m^−2^ s^−1^, indicating an initial positive response of photosynthesis to higher water availability. In the flowering stage, regimes T2 and T4 were 1.3- and 1.2-fold higher than T1, with a Pn of 12 and 11 µmol CO_2_ m^−2^ s^−1^, respectively (Figure 3B). However, during the fruiting stage, a significant increase in Pn was observed, especially in T3 (Figure 3B). Bayesian analysis confirmed the significance of the variability concerning phenological stage and irrigation regime on Pn, highlighting that while T2 initially increased photosynthesis compared to T1, both treatments later experienced declines, suggesting negative effects of excessive irrigation. On the other hand, T3 and T4 showed improvements in later stages, indicating the benefits of more moderate water applications.

Stomatal conductance (gs) also varied significantly between the different treatments and phenological stages, as shown in Figure 3C,D. In both seasons, an increase in gs values was observed in all regimes at fruiting. Specifically, the T2 treatment had the maximum gs value of 0.14 mmol H_2_O m^−2^ s^−1^ during fruiting for the 2021/2022 season, while the highest conductance values were recorded during fruiting in T2 and T3, reaching 0.36 and 0.35 mmol H_2_O m^−2^ s^−1^, respectively, in the 2022/2023 season.

Transpiration (E) differed significantly (*p* < 0.001) between treatments and phenological stages, as illustrated in Figure 3E,F. For the 2021/2022 season, a similar dynamic to that reported for gs was observed during all three physiological stages evaluated. However, for the 2022/2023 season, during flowering an increase in E was recorded in all treatments, especially in T2, T3, and T4, with values reaching 5.9, 5.5, and 5.7 mmol H_2_O m^−2^ s^−1^, respectively.

**Figure 4:**
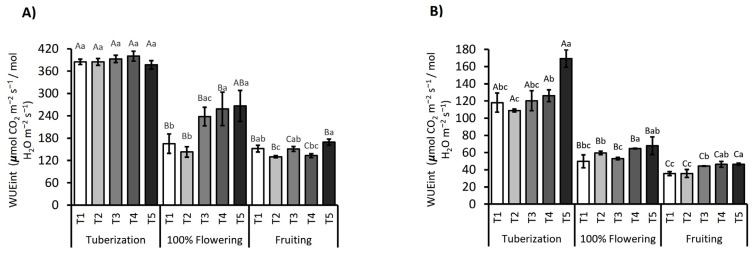
Intrinsic water use efficiency (WUE_int_) in the *S. tuberosum* var. Puyehue-INIA during the 2021/2022 (**A**) and 2022/2023 (**B**) growing seasons. Plants were subjected to five irrigation regimes: well-watered (T1, 100%), over-irrigated (T2, 130%), and deficit irrigation treatments (T3, 75%; T4, 50%; and T5, 30%). Measurements were conducted at three phenological stages: tuberization, peak flowering, and fruiting. Bars represent the mean ± standard error (SE). The numbers above bars denote the percentage change in WUE_int_ relative to the fully irrigated control (T1). Different capital letters indicate statistically significant differences between treatments across phenological stages, whereas lowercase letters indicate differences within each stage, according to Tukey’s HSD test (*p* ≤ 0.05).

Regarding intrinsic water use efficiency (WUE_int_), for the 2021/2022 season, ANOVA analysis showed that the phenological stage and the influence of irrigation treatments and their interaction with the phenological stage had a significant effect (*p* < 0.001; Figure 4A). Additionally, Bayesian analysis provided further evidence of the importance of the temporal factor, where treatments explained 47% and the interaction between treatments and the phenological stage explained 52% of the variation. For the 2022/2023 season, a similar pattern was observed, with significant variation (*p* < 0.001) in WUE_int_ values influenced by treatments, the interaction of the phenological stage, and the irrigation treatment. During the 2021/2022 tuberization stage, WUE_int_ remained high and consistent across all irrigation treatments. However, for the 2022/2023 season, potatoes under optimal irrigation conditions (T1) and over-irrigation (T2) showed lower WUE_int_ values compared to those under water deficit conditions for the three phenological stages, with T5 increasing by 1.36-fold compared to T1 (Figure 4B). However, during the flowering phase, a significant decrease in WUE_int_ was detected in all treatments, with T1 experiencing a 57% reduction compared to tuberization in the first season (Figure 4A). In contrast, during the 2022/2023 season, WUE_int_ values in treatments T5 (30% optimal irrigation) and T4 increased by 1.36 and 1.29-fold compared to T1, with values of 68 and 65 µmol CO_2_ m^−2^ s^−1^/mol H_2_O m^−2^ s^−1^, indicating greater WUE_int_ under these deficit irrigation regimes. During the fruiting phase, a significant reduction was observed for all treatments compared to flowering and tuberization. However, again, well-irrigated and over-irrigated treatments were significantly lower than treatments under water deficit conditions.

### 3.4. Photosynthetic Pigments

Chlorophylls: During each season, pigment concentrations were chiefly driven by developmental stage (two-way ANOVA, stage *p* < 0.001; treatment *p* < 0.05). During tuberization, Chl *a* ranged from 0.52 to 0.64 mg g^−1^ DW and Chl *b* from 0.22 to 0.26 mg g^−1^ DW (Table 2), with T1 and T2 at the upper bounds. At peak flowering, both chlorophylls declined by ≈15% in well-watered plants and by up to 25% in the severe deficit treatments (T4, T5). A further drop of 30–40% occurred at fruit set, leaving T4 and T5 with the lowest values (Chl *a* ≈ 0.33–0.44; Chl *b* ≈ 0.18–0.19 mg g^−1^ DW). Seasonal conditions modulated these trends: mean chlorophylls in 2022/2023 were ~10% lower than those in 2021/2022, consistent with the hotter, drier year. The stage × treatment interaction was modest (*p* = 0.08), indicating that irrigation chiefly shifted the magnitude, but not the temporal pattern, of chlorophyll depletion.

Carotenoids: Baseline carotenoid content at tuberization was ≈1.10–1.20 mg g^−1^ DW in T1, but water deficit triggered significant over-accumulation. Specifically, T4 and T5 surpassed the control by 15–30% at tuberization and by 40–50% at flowering (peak values ≤ 1.53 mg g^−1^ DW). At fruit set, carotenoids in T4–T5 remained ~20% above T1, whereas this parameter continued to decline in well-watered regimes. Consequently, the Car:Chl ratio increased monotonically with irrigation deficit, implying a shift from light harvesting to photoprotective pigmentation under water stress.

### 3.5. Yield

**Figure 5:**
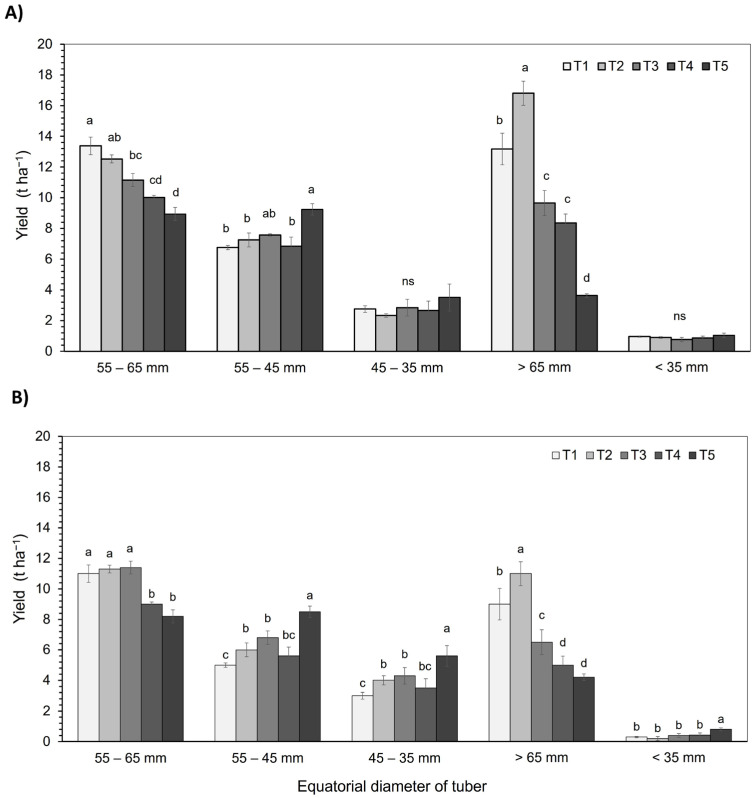
Yield (t ha^−1^) of the *S. tuberosum* var. Puyehue-INIA during the 2021–2022 (**A**) and 2022–2023 (**B**) growing seasons. Plants were subjected to five irrigation regimes: well-watered (T1, 100%), over-irrigated (T2, 130%), and deficit irrigation treatments (T3, 75%; T4, 50%; and T5, 30%). Yield was classified according to tuber equatorial diameter into commercial size (55–65 mm), seed category 1 (55–45 mm), seed category 2 (45–35 mm), larger than commercial size (>65 mm), and non-commercial size (<35 mm). Bars represent the mean ± standard error (SE). Different lowercase letters indicate statistically significant differences among treatments within the same size category according to Tukey’s HSD test (*p* ≤ 0.05).

The results obtained in both seasons show significant differences (*p* < 0.05) in tuber yield (t ha^−1^) between treatments and the different size categories (Figure 5). In the commercial size category (55–65 mm), during the 2021/2022 season, T1 achieved the highest yield (13.4 t ha^−1^), followed by T2, with a progressive decrease in T3, T4, and T5. However, in the 2022/2023 season, the highest yield in this category was recorded in T3, along with T2 and T1. The treatment with the greatest water deficit (T5) showed a significant increase in seed category 1 (45–55 mm) in both seasons, with yields 1.3- and 1.5-fold higher than the control (T1), respectively. On the other hand, in the category of tubers larger than the commercial size (>65 mm), T2 presented the highest yields in both seasons, although with more pronounced reductions in T3, T4, and T5, especially in the treatment with the greatest water deficit (T5), where a decrease of up to 64% was observed in 2022/2023.

## 4. Discussion

The analysis of the physiological responses of *S. tuberosum* under different deficit irrigation strategies provides a deeper understanding of how these practices affect water use efficiency, photosynthesis, and other critical parameters. 

### 4.1. Physiological Responses and Meteorological Conditions

The physiological responses to drought in *S. tuberosum* significantly depend on the variety and the seed source [18,36,37]. In this study, it was observed that stomatal conductance (gs) decreased under water stress conditions, a water conservation mechanism that has been widely documented [38,39]. This decrease in gs is an efficient strategy to minimize water loss, aligning with previous studies [40,41,42,43,44]. 

The variability in water stress response between deficit irrigation regimes also underscores genotype-dependent drought tolerance in potato. The observed reduction in dry matter yield under water limitation agrees with earlier reports of similar negative effects [20,45]. Nevertheless, several treatments preserved a high water use efficiency (in this study WUE = WUE_int_, the ratio of net CO_2_ assimilation Pn to stomatal conductance g_s_), indicating that breeding for elevated WUE _int_ remains a promising avenue to enhance drought resilience. Comparable behavior has been described for *Solanum lycopersicum*, where controlled deficit irrigation at 65–75% ETc increased WUE_int_ by 4–12% without compromising ΦPSII or the marketable yield of tomatoes [46]. However, the magnitude of the response is species-specific: tomato can sustain Pn at soil relative water contents down to ≈45%, whereas in our study, potato exhibited a marked decline below ≈60% SRWC, reflecting its shallower root system and more anisohydric stomatal regulation [47]. Notably, both crops belong to the Solanaceae family and exhibit broadly comparable seasonal crop water requirements (≈500–700 mm for a 120–150-day cycle), reinforcing the physiological analogy between the two species [48,49]. In tomato, higher vapor pressure deficit (VPD) environments raise WUE_int_ by restricting gs while maintaining Pn, and soil water deficit induces earlier stomatal closure at high VPD, pointing to an abscisic acid-independent mechanism that bolsters drought tolerance [50]. These inter-specific contrasts emphasize that water savings above 25% ETc are feasible in tomato but may exceed the physiological safety margin in potato.

### 4.2. Gas Exchange and Photosynthetic Pigments

Across both seasons, PSII performance, pigment pools, and WUE_int_ displayed a coherent, stage-dependent response to the irrigation gradient. During tuberization, Fv’/Fm’ (≈0.70) and ΦPSII were stable across all water regimes, indicating a resilient photochemical apparatus under mild stress. At flowering and fruit set, both parameters declined sharply (−15–25%), with the steepest drops in the severe deficit treatments (T4, T5), but also in the over-irrigated treatment (T2), where ETR fell by ~20%, suggesting photoprotective down-regulation under both water excess and shortage [51,52,53,54,55] 

These functional shifts paralleled pigment dynamics. Chlorophylls decreased progressively from tuberization to fruit set, reflecting either stress-induced degradation or dilution by leaf expansion. Notably, T4 (flowering) and T5 (fruiting) retained higher chlorophyll on a per mass basis, likely a concentration effect linked to reduced leaf area rather than de novo synthesis [4,56]. In contrast, carotenoids rose by 15–50% under moderate-to-severe deficit, elevating the Car:Chl ratio and providing additional non-enzymatic antioxidant capacity against ROS generated under drought and high irradiance [57,58]. The concomitant fall in Fv′/Fm′ and rise in carotenoids in T4–T5 (Figure 2) is consistent with a photoprotective rather than a photoinhibitory response [59,60,61]. 

Gas exchange data close the loop: Net photosynthesis (Pn) tracked the decline in chlorophyll, while stomatal conductance fell more steeply, such that WUE_int_ remained high during tuberization but dropped by 30–40% at flowering, the stage of maximal atmospheric demand and pigment turnover [31,32]. At T5 (30% irrigation), stomatal conductance declined by 62% and net photosynthesis by 48% relative to the control, yet intrinsic water use efficiency rose 26% as a consequence of strong stomatal limitation. During fruit set, partial stomatal reopening improved WUEᵢₙₜ without fully restoring Pn, simultaneously with carotenoid-based photoprotection. These responses reveal an integrated acclimation in which potato leaves coordinate pigment stoichiometry, electron transport, and stomatal behavior to balance carbon gain, water conservation, and photoprotection across developmental stages and contrasting water supplies.

### 4.3. Principal Component Analysis (PCA)

**Figure 6:**
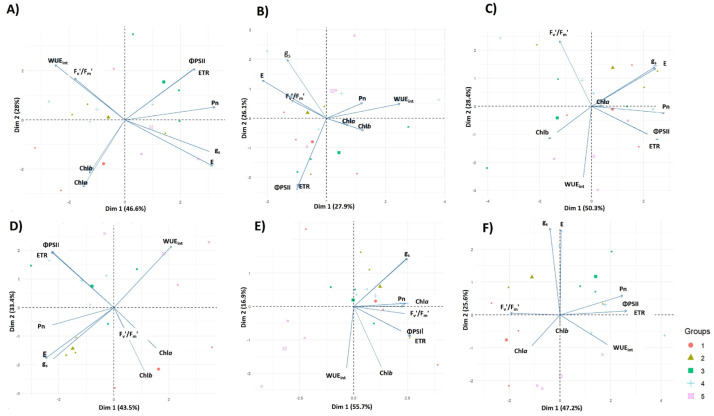
Principal component analysis (PCA) of physiological parameters in the *S. tuberosum* var. Puyehue-INIA during the 2021/2022 (**A**–**C**) and 2022/2023 (**D**–**F**) growing seasons under different irrigation regimes: well-watered (1, 100%), over-irrigated (2, 130%), and deficit irrigation treatments (3, 75%; 4, 50%; and 5, 30%). The graphs correspond to three phenological stages: tuberization (**A**,**D**), peak flowering (**B**,**E**), and fruiting (**C**,**F**). Arrows represent the contribution of physiological variables to the model, including net photosynthesis rate (Pn), stomatal conductance (gs), transpiration rate (E), quantum efficiency of photosystem II (ΦPSII), maximum photochemical efficiency (Fv’/Fm’), intrinsic water use efficiency (WUE_int_), electron transport rate (ETR), and chlorophyll content (Chla and Chlb).

In the 2021–2022 season, PC1 and PC2 explained 74.64% of the total variability (PC1 = 46.61%, PC2 = 28.03%), indicating that most of the dataset structure could be effectively represented in a two-dimensional space (Figure 6A–C). Treatment groups exhibited distinct clustering patterns, with T1 (100% irrigation) and T2 (130% irrigation) positioned in the region associated with higher values of net photosynthesis (Pn) and stomatal conductance (gs), consistent with greater water availability. Conversely, T4 (50% irrigation) and T5 (30% irrigation) were located in distant areas, suggesting distinct physiological adjustments characterized by lower assimilation rates but increased water use efficiency (WUE_int_). The intermediate treatment, T3 (75% irrigation), was positioned between these two extremes, reinforcing its transitional nature in response to different water availability conditions.

Regarding the contribution of physiological traits, PC1 was mainly associated with Pn, gs, and transpiration (E), highlighting the strong interdependence between gs, water loss, and CO_2_ fixation. Meanwhile, PC2 captured the variability related to Fv’/Fm’, WUE_int_, and, to a lesser extent, chlorophyll content (Chl*a*, Chl*b*). Notably, the opposing positioning of WUE_int_ relative to gs and E confirmed the trade-off between maximizing photosynthetic performance and conserving water. The clear differentiation between treatments suggests the existence of two contrasting physiological strategies: under well-watered conditions, plants exhibit higher carbon assimilation and stomatal opening, whereas under restricted irrigation, photosynthetic activity declines, but water use efficiency improves.

In the 2022–2023 season, the first two principal components explained 76.9% of the total variance (PC1 = 43.5%, PC2 = 33.4%; Figure 6D–F). Similar to the first season, T1 and T2 clustered closely together, indicating similar physiological responses under higher water availability. In contrast, T4 and T5 formed a distinct group, suggesting that water restriction induced comparable physiological effects in these treatments. T3 (75% irrigation) remained in an intermediate position, strengthening its role as a transitional treatment between high- and low-irrigation groups.

The variable contribution to PC1 and PC2 was consistent with the previous season. PC1 was predominantly associated with gs, E, and Pn, confirming the tight relationship between stomatal conductance, transpiration, and CO_2_ assimilation. Additionally, ΦPSII and electron transport rate (ETR) exhibited a strong correlation and contributed significantly to this principal component. In PC2, WUE_int_ was positioned in the opposite direction to g_s_ and E, supporting the observation that increased stomatal conductance and transpiration reduce WUE_int_. Furthermore, Chl*a* and Chl*b* aligned with this second component, suggesting that their variability was not directly related to photosynthetic performance or water use efficiency under these irrigation treatments.

These results demonstrate consistent physiological responses between seasons, reinforcing the trade-offs between maximizing photosynthesis and optimizing water use under different irrigation regimes during the tuberization stage. The clear clustering of treatments suggests that strategic water management can indeed drive distinct physiological adaptations, influencing both carbon assimilation and water conservation in potato crops.

The PCA performed for each phenological stage and season yielded a highly consistent structure. In all six cases, the first two components explained between 55% and 70% of the total variance, and inclusion of a third axis raised the cumulative explanation above 80%. PC1 invariably opposed gs and E to WUE_int_, capturing the fundamental trade-off between maximizing CO_2_ uptake and minimizing water loss. PC2 grouped photochemical performance (ΦPSII, ETR, Fv’/Fm’) with chlorophyll *a* and *b*, indicating that variations in light harvesting and electron transport occurred largely independently of stomatal regulation. Carotenoid loadings shifted towards the WUE_int_ vector under deficit irrigation, consistent with their photoprotective role when CO_2_ diffusion is curtailed.

Treatment scores projected cleanly onto this framework. Well-watered and over-irrigated plots (T1, T2) clustered on the high-gs side of PC1, whereas moderate and severe deficits (T4, T5) migrated towards the WUE_int_/carotenoid quadrant; the intermediate treatment (T3) occupied an intermediate position. Superimposed phenological effects were evident; at flowering and fruit set, every treatment shifted downward along PC2, reflecting the universal decline in photochemical efficiency and chlorophyll content documented earlier. These patterns confirm that stomatal control was the primary axis of physiological divergence between irrigation regimes, while pigment adjustments and PSII tuning constituted a secondary, stage-driven layer of variability (Figure 6A–F).

### 4.4. Correlation Matrix

**Figure 7:**
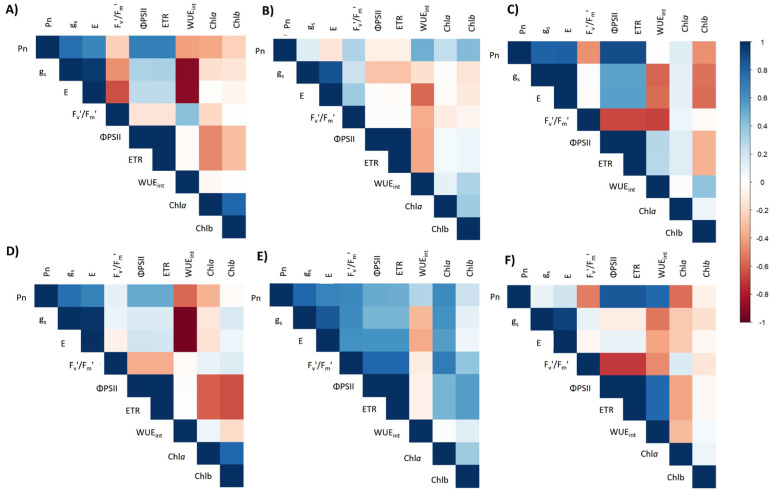
Correlation matrix of physiological parameters in the *S. tuberosum* var. Puyehue-INIA during the 2021/2022 (**A**–**C**) and 2022/2023 (**D**–**F**) growing seasons under different irrigation regimes: well-watered (T1, 100%), over-irrigated (T2, 130%), and deficit irrigation treatments (T3, 75%; T4, 50%; and T5, 30%). The heatmaps correspond to three phenological stages: tuberization (**A**,**D**), peak flowering (**B**,**E**), and fruiting (**C**,**F**). The color scale represents Pearson’s correlation coefficients between physiological variables, where dark blue indicates strong positive correlations, dark red indicates strong negative correlations, and lighter shades reflect weaker associations. The analyzed variables are net photosynthesis rate (Pn), stomatal conductance (gs), transpiration rate (E), quantum efficiency of photosystem II (ΦPSII), maximum photochemical efficiency (Fv’/Fm’), intrinsic water use efficiency (WUE_int_), electron transport rate (ETR), and chlorophyll content (Chla and Chlb).

During the 2021/2022 season, analysis of the 28 pairwise combinations yielded 15 significant positive and 13 significant negative correlations (|r| ≥ 0.50, *p* < 0.05; Figure 7A–C). The strongest positive link occurred between the effective quantum yield of PSII and the electron transport rate (ΦPSII ETR, r ≈ +0.99), confirming that any change in photochemical efficiency was mirrored almost instantaneously in electron flow. At the opposite extreme, intrinsic water use efficiency was tightly and negatively coupled to stomatal conductance (WUE_int_-gs, r ≈ –0.91), underscoring the water cost of maintaining stomata open.

The hotter and drier 2022/2023 season displayed a similar structure, with 16 positive and 12 negative correlations (Figure 7D–F). Once again, Φ-PSII and ETR were virtually superimposed (r ≈ +0.99), highlighting the robustness of their functional coupling across contrasting environments. The trade-off between carbon assimilation and water conservation intensified, as reflected by an even stronger negative correlation between WUE_int_ and gs (r ≈ –0.95). These two variable pairs (ΦPSII-ETR and WUE_int_-gs) thus encapsulate the core physiological tension observed in both years: PSII efficiency governed electron transport, while stomatal regulation determined the balance between photosynthetic gain and hydric economy.

### 4.5. Multiple Linear Regression Models

Multiple regression analysis confirmed that Pn was chiefly governed by the joint action of gs and ΦPSII, whereas the direct contribution of E was minor and stage dependent. In 2021/2022, the model explained 79% of Pn variance at tuberization and 94% at fruit set; in both cases, ΦPSII emerged as the dominant positive predictor, while gs became influential only when water supply was ample. Variance inflation factors (VIF) ranged from 1.22 to 1.47, ruling out multicollinearity between gs, E, and ΦPSII. Thus, model estimates remained valid and required no re-specification. During peak flowering, the equation lost power (r^2^ ≈ 0.37) and individual coefficients for gs and E changed sign, suggesting that additional factors, likely metabolic or micro-environmental, limit assimilation at this peak demand stage.

In the drier 2022/2023 season, the same set of predictors accounted for 84% of Pn variability at tuberization and 76% at fruit set. Here, gs gained statistical weight alongside ΦPSII in early growth, but E exerted a small negative influence, indicating that excess water loss did not translate into higher carbon gain. At flowering, the model fit improved relative to 2021/2022 (r^2^ ≈ 0.66), yet only gs remained significant, again pointing to stomatal regulation as the principal lever of photosynthetic adjustment when atmospheric demand peaks.

Taken together, these regressions highlight a consistent hierarchy: Photochemical efficiency sets the ceiling for carbon assimilation, stomatal conductance modulates that potential according to water availability, and transpiration per se seldom enhances Pn once gs is accounted for. Including additional descriptors of internal water status or biochemical capacity may further refine predictions, but the current models capture the essential physiological controls operating across different irrigation regimes and developmental stages.

### 4.6. Hierarchical Clustering Analysis (HCA)

**Figure 8:**
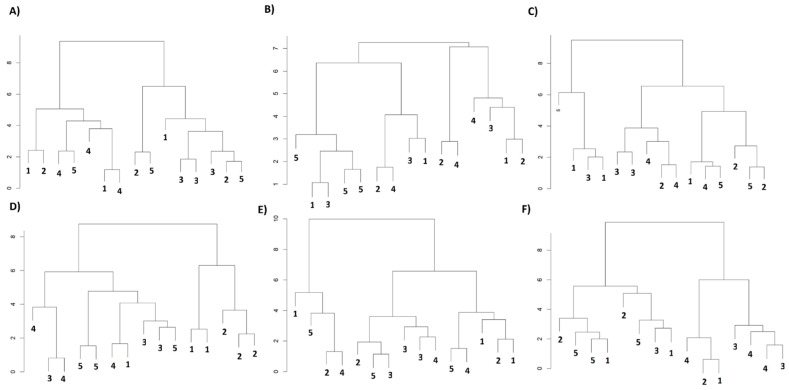
Hierarchical clustering analysis (HCA) of physiological parameters in the *S. tuberosum* var Puyehue-INIA during the 2021/2022 (**A**–**C**) and 2022/2023 (**D**–**F**) growing seasons under different irrigation regimes: well-watered (1, 100%), over-irrigated (2, 130%), and deficit irrigation treatments (3, 75%; 4, 50%; and 5, 30%). Dendrograms were constructed using Euclidean distances and Ward’s minimum variance method (Ward.D2). Panels correspond to the three evaluated phenological stages: tuberization (**A**,**D**), peak flowering (**B**,**E**), and fruiting (**C**,**F**). The height of the branches represents the degree of dissimilarity among treatments, with shorter branches indicating greater physiological similarity. Clustering patterns reflect distinct physiological strategies adopted by plants in response to water availability, based on gas exchange, photosynthetic performance, and water use efficiency traits.

In every phenological stage, hierarchical clustering confirmed the dichotomy already revealed by PCA and correlation analysis (Figure 8). During tuberization (both seasons) the dendrogram split the five irrigation treatments into two coherent groups: a “high-water” cluster (T1-T2) with greater CO_2_ assimilation and a “water-limited” cluster (T4-T5) characterized by lower photosynthesis but higher WUE_int_. T3 consistently occupied an intermediate branch, marking the physiological threshold between the two strategies.

At peak flowering the structure became looser, especially in 2021/2022, when T1, T2, and T3 were intermingled while the deficit treatments remained distinct. This dispersion implies that at the peak of atmospheric demand, additional factors (temperature spikes, micro-site variation, or metabolic constraints) blur the separation imposed solely by irrigation. The drier 2022/2023 season restored a clearer two-cluster pattern, yet Euclidean distances indicated a gradient rather than a sharp boundary, again placing T3 midway along that continuum.

During fruit set, the classification tightened in both years: One subtree combined high gs and E (assimilation-driven strategy), while the other grouped low gs with elevated WUE_int_ (conservation strategy). Euclidean distances remained widest between these subtrees, underscoring the stability of the dual adaptive modes as the canopy aged. Altogether, the HCA corroborates a consistent hierarchy of responses in that well-watered plants maximized carbon gain, severely restricted plants economized water, and the 75% treatment (T3) straddled the switch point between both programs.

The multivariate approach therefore moves the analysis beyond sole reliance on *p*-values and highlights that coherent physiological patterns link the irrigation gradient to biochemical photoprotection and water use efficiency. In both seasons, the concerted displacement of the carotenoid and WUE_int_ vectors opposite to g_s_ and E along PC1 denoted a coordinated shift from an “assimilation” to a “conservation” strategy as water deficit intensified, echoing the re-direction of carbon flux toward isoprenoid pathways reported in potato and other solanaceous crops under drought [62,63]. The 15–50% increase in carotenoids detected in T4–T5 serves not only as an energy sink and ROS quencher but also as a commercially desirable trait, enhancing provitamin-A content and color stability in tubers destined for processing [64,65]. Cultivars enriched in β-carotene, for instance, retain greater post-harvest firmness and show less browning during frying when grown under controlled deficit irrigation, opening prospects for joint water-saving and biofortification programs [66].

The dendrogram topology has immediate agronomic relevance: the consistent separation of T1–T2 (high g_s_, high Pn, low Car:Chl) from T4–T5 (low g_s_, high Car:Chl, high WUE_int_) establishes a physiological threshold (~75%) beyond which the crop switches from carbon gain to water conservation mode. The intermediate positioning of T3 confirms that a moderate deficit can trigger photoprotection and improve water use efficiency without incurring severe yield penalties, in agreement with studies reporting 20–30% gains in water use efficiency when potatoes are irrigated at 70–80% or subjected to partial root zone drying [67,68]. Together, these findings reinforce the view that carotenoid–stomatal adjustment is an integrative marker of functional plasticity in potato, offering a lever to reconcile productivity, nutraceutical quality, and water savings in the context of climate change.

### 4.7. Yield

The reduction in yield under water deficit conditions could be related to several physiological and environmental factors [69,70]. In addition to decreases in source activity, deficit irrigation elicits plastic responses that can buffer yield penalties: potatoes subjected to moderate soil drying have greater root length densities and penetration depths, thereby sustaining water uptake from subsoil horizons and stabilizing canopy water status [71]. They also accumulate compatible solutes (proline, soluble sugars, raffinose) that lower cellular osmotic potential and maintain turgor-driven assimilate transport [72,73], and the resulting increase in xylem-borne abscisic acid fine-tunes stomatal aperture, raising intrinsic water use efficiency without severely restricting CO_2_ diffusion [74]. Concomitant with these adjustments, carotenoid concentrations in leaves and tubers increased by 15–50% under the most restrictive treatments (T4, T5), a response consistent with the drought-induced rise in total carotenoid content reported for several cultivars (e.g., an average rise of 22% in “Marabel” and “Laura”) after 71 days of controlled water deficit [62]. This enrichment augments non-photochemical quenching capacity, limits singlet oxygen formation, and has direct commercial relevance, as higher lutein and violaxanthin contents improve tuber color stability during processing and contribute provitamin A equivalents [64]. Indeed, breeding material selected for elevated β-carotene retains fry color and antioxidant capacity even under regulated deficit irrigation, highlighting an opportunity to couple water-saving strategies with nutritional biofortification goals [63].

A greater number of tubers per plant has been observed when limited water availability is combined with more frequent irrigation, and yield losses have also been linked to decreased tuber size, which is partially compensated by an increased tuber number per plant [75]. In our study, the highest soil moisture regime (T2) produced a significantly greater total tuber yield than the lower irrigation regimes, yet the moderate deficit treatment (T3, 75%) maintained commercial yield, suggesting that the aforementioned root, osmotic, and hormonal adjustments mitigated the reduction in water supply. These results are consistent with previous studies that reported comparable gains under similar watering thresholds [76,77,78]. Declines in tuber weight under water stress have likewise been associated with reductions in leaf area and photosynthesis per unit leaf area [79,80]. 

Tuber yield is strongly influenced by irrigation level; for example, a 20% reduction in water supply caused a 9.3% decrease in the “Tomba” variety, while 40% and 60% reductions led to yield losses of 25.7% and 42.7%, respectively, in the “Cara” variety [81,82]. Implementing slight irrigation restrictions during the early stages of cultivation has been shown to improve drought resistance, conserve water, and prevent severe yield reductions [83].

This effect on potato tuber yield may stem from genetic variability and the contrasting soil and climatic conditions where the crop is grown [84]. Over-irrigation, by contrast, can intensify nutrient leaching, waterlogging, pest and disease incidence, and the operational and maintenance costs of the irrigation system [85,86]. Consequently, optimizing water application reduces production costs, enhances plant growth, and improves crop yield. 

### 4.8. Seasonal Climate Variability and Transferability of the 75% ETc Threshold

The marked climatic disparity between effective precipitation of 180 mm in 2021/2022 versus 85 mm in 2022/2023 (−53%) and ET₀ peaks of 6.3 mm d^−1^ versus 7.8 mm d^−1^, respectively, provided a natural test of the robustness of the adopted replenishment threshold. Even under higher atmospheric aridity and a mean VPD that rose to 0.78 kPa during flowering in the second season, the treatment that replenished 75% (T3) retained both net photosynthetic rate and marketable caliber, confirming the physiological resilience of “Puyehue-INIA” and the water buffering capacity conferred by the local Andisol (θ FC ≈ 0.52 m^3^ m^−3^).

Recent meta-analyses place this threshold among the most efficient strategies: the synthesis of 108 field trials in China showed 10–32% increases in WUE with 70–80% ETc replenishment, with a yield sacrifice of only 16% compared to full irrigation [66]. In temperate humid latitudes (Prince Edward Island, Canada), hydrological simulations reveal that seasonal rainfall ≥ 150 mm is sufficient to supplement at 70–75% ETc and thus balance annual water amounts, while years < 100 mm require increasing replenishment to 80–85% to avoid soil deficits and caliber penalties [87]. Long-term trials in semiarid environments also warn that in exceptionally dry seasons, replenishment to 80% ETc maintains yield, but values ≤ 70% generate aerial biomass losses of up to 20% [88]. These results are consistent with decreases of 15–40% observed when replenishment drops to 60–65% ETc in continental climates [89].

The interaction between atmospheric demand and soil water-holding capacity thus emerges as a key transfer criterion. In deep Andisols (TAW ≈ 150 mm) and VPD < 1.2 kPa, the 75% ETc set point remains safe even with only 80 mm of rainfall before flowering. In contrast, in sandy loam soils (TAW < 90 mm) or when ET₀ peaks exceed 7.5 mm d^−1^, the literature recommends adjusting the threshold to ≥80% ETc or adopting PRD/SDI supplemented with thermal sensors in the soil to avoid losses in tuber filling [90]. These findings underscore that the proposed strategy has broad applications provided it (i) integrates effective rainfall prior to flowering, (ii) forecasts peak VPD, and (iii) profiles storage capacity in decision-making models for regulated deficit irrigation.

## 5. Conclusions and Future Prospects

This research set out to delineate deficit irrigation thresholds that rationalize water use in *Solanum tuberosum* without compromising tuber yield. Over two contrasting seasons, we confirmed that replenishing ≈ 75% of crop demand, equivalent to a reduction of about 80 mm ha^−1^ relative to full irrigation, sustains net photosynthesis and stomatal conductance through the sensitive phases of tuberization and peak flowering, maintains intrinsic water use efficiency (WUEᵢₙₜ) at profitable levels, and delivers tuber yields statistically indistinguishable from the fully irrigated control. In economic terms, the reduction of 1.9 kg m^−3^ of water required under this regime translates directly into lower pumping costs and a reduced environmental footprint for growers who pay volumetric water tariffs. By contrast, harsher deficits (50% or 30%) imposed sizable yield penalties, especially when they coincided with phenologically demanding stages, while surplus irrigation (130%) afforded no tangible yield advantage and diluted overall WUEᵢₙₜ. Tuberization and flowering therefore emerge as the periods that most merit full or near-full water supply, whereas fruit set proves more tolerant of moderate restriction, opening the door to strategic re-allocation of scarce irrigation resources.

Although the 75% benchmark performed robustly in our environment, its feasibility is conditional on site-specific edapho-climatic and operational factors and should not be adopted as a universal prescription. The trials were run on a well-drained volcanic Andisol whose high porosity minimized anoxia; on finer-textured Alfisols or Vertisols the same threshold could induce transient waterlogging, whereas on coarse Arenosols it could have triggered earlier water deficit. Taken together, the evidence confirms that judicious allocation of water to tuberization and flowering, while permitting minor deficits during phenologically tolerant windows, offers a pragmatic balance between productivity and resource sustainability.

Future research should extend these thresholds to a wider range of cultivars and climates, giving priority to genotypes with intrinsically high WUEᵢₙₜ. Integrating real-time diagnostic soil moisture probes, sap flow sensors, and on-canopy micrometeorology will allow for dynamic adjustment of irrigation to meet physiological demand. Because this study was limited to the single cultivar “Puyehue-INIA,” multi-site trials covering several varieties and maturity groups are required to assess the robustness of the proposed benchmark of 75% of demand.

## Figures and Tables

**Table 1:** Morphological parameters, applied T1 irrigation, and effective precipitation from emergence to senescence in potato crops under irrigation conditions during the peak demand period for the 2021/2022 and 2022/2023 seasons.

	2021/2022 Season			2022/2023 Season		
Phenological State	Height	Irrigation	Effective Rainfall	Height	Irrigation	Effective Rainfall
	(m)	(mm)	(mm)	(m)	(mm)	(mm)
Pre-emergence	0	20.0	0	0	20.0	7.1
Emergence	0.1	35.2	0	0.1	32.0	0
Vegetative development	0.21	28.6	0	0.22	32.0	27.2
Tuber initiation	0.45	19.2	26.1	0.4	36.3	0
Flowering initiation	0.67	45.8	26.1	0.5	47.3	7.0
Flowering	0.57	49.3	0	0.56	55.2	0
Fruit formation	0.4	53.1	11.32	0.5	46.0	0
Fruit and seed maturation	0.45	0	0	0.5	13	0
Senescence	0	0	0	0	0	0
Total		251.2	63.5		281.5	41.3
**Total water**		314.7			322.8	

**Table 2:** Chlorophyll *a*, chlorophyll *b*, and carotenoids of the Puyehue-INIA variety (S. *tuberosum*) during the 2021/2022 and 2022/2023 seasons, subjected to well-watered conditions (T1, 100%), over-irrigated conditions (T2, 130%), and deficit irrigation conditions (T3, 75%; T4, 50%; and T5, 30%) for the phenological periods (tuberization), peak flowering, and fruiting. Uppercase letters indicate differences between treatments in different harvests, and lowercase letters show differences between treatments in the same harvest according to Tukey’s test (*p* ≤ 0.05).

Treatment	Collection of Plant Material (2021/2022)					
	Tuberization		Peak Flowering		Fruiting	
	Average	Standard Error	Average	Standard Error	Average	Standard Error
Chlorophyll *a* content (mg g^−1^ DW)						
T1	0.64 ± 0.02 Aa		0.55 ± 0.02 Bab		0.44 ± 0.05 Cab	
T2	0.55 ± 0.01 Ab		0.55 ± 0.03 Aab		0.45 ± 0.03 Ba	
T3	0.52 ± 0.01 Ab		0.54 ± 0.01 Aab		0.35 ± 0.05 Bbc	
T4	0.55 ± 0.00 Ab		0.58 ± 0.02 Aa		0.33 ± 0.07 Bc	
T5	0.57 ± 0.02 Aab		0.48 ± 0.01 Bb		0.44 ± 0.02 Bab	
Chlorophyll *b* content (mg g^−1^ DW)						
T1	0.26 ± 0.00 Aa		0.22 ± 0.01 ABa		0.19 ± 0.02 Ba	
T2	0.24 ± 0.01 Aab		0.22 ± 0.01 ABa		0.18 ± 0.01 Ba	
T3	0.22 ± 0.01 Ab		0.21 ± 0.01 Aa		0.21 ± 0.05 Aa	
T4	0.22 ± 0.01 Aab		0.21 ± 0.01 Aa		0.16 ± 0.01 Ba	
T5	0.23 ± 0.00 Aab		0.22 ± 0.01 Aa		0.19 ± 0.00 Bab	
Carotenoid content (mg g^−1^ DW)						
T1	1.16 ± 0.04 Aa		1.08 ± 0.07 Aba		0.87 ± 0.05 Bab	
T2	1.03 ± 0.03 Aa		1.05 ± 0.07 Aab		0.88 ± 0.03 Aa	
T3	0.99 ± 0.03 Aa		1.09 ± 0.04 Aa		0.77 ± 0.05 Bab	
T4	0.95 ± 0.10 Aa		1.14 ± 0.05 ABa		0.64 ± 0.07 Bb	
T5	1.05 ± 0.01 Aa		0.93 ± 0.06 Ab		0.88 ± 0.02 Bab	
Collection of plant material (2022/2023)						
Chlorophyll *a* content (mg g^−1^ DW)						
T1	0.65 ± 0.050 Ba		0.70 ± 0.027 Aa		0.60 ± 0.049 Ba	
T2	0.60 ± 0.019 Bab		0.65 ± 0.040 Aab		0.55 ± 0.029 Ba	
T3	0.62 ± 0.012 Ba		0.68 ± 0.013 Aa		0.58 ± 0.049 Ba	
T4	0.58 ± 0.017 Ab		0.60 ± 0.030 Abc		0.52 ± 0.063 Aab	
T5	0.50 ± 0.041 ABc		0.55 ± 0.020 Ac		0.45 ± 0.028 Bb	
Chlorophyll *b* content (mg g^−1^ DW)						
T1	0.30 ± 0.021 Ba		0.35 ± 0.010 Aa		0.28 ± 0.019 Ba	
T2	0.28 ± 0.019 Abab		0.32 ± 0.017 Ab		0.25 ± 0.016 Bab	
T3	0.30 ± 0.019 Aa		0.33 ± 0.018 Aab		0.27 ± 0.023 Aab	
T4	0.27 ± 0.010 Bb		0.30 ± 0.009 Ab		0.24 ± 0.019 Bb	
T5	0.22 ± 0.009 Bc		0.25 ± 0.009 Ac		0.20 ± 0.018 Bc	
Carotenoid content (mg g^−1^ DW)						
T1	1.20 ± 0.039 Ab		1.30 ± 0.068 Abc		1.15 ± 0.049 Ab	
T2	1.15 ± 0.025 Ab		1.20 ± 0.065 Ac		1.20 ± 0.035 Ab	
T3	1.25 ± 0.031 Bab		1.40 ± 0.035 Ab		1.20 ± 0.051 Bb	
T4	1.30 ± 0.099 Bab		1.45 ± 0.047 Aab		1.35 ± 0.072 ABa	
T5	1.30 ± 0.009 Ba		1.50 ±0.063 Aa		1.40 ± 0.018 Ba	

## Data Availability

The datasets generated and/or analyzed during the current study are not deposited in a public repository but are available from the corresponding authors upon reasonable request. Researchers interested in accessing the underlying data, or in exploring future collaborations, should contact the corresponding authors at: Dr. Rafael López-Olivari—rafael.lopez@inia.cl; Dr. Claudio Inostroza-Blancheteau—claudio.inostroza@uct.cl; All requests will be considered in the context of ethical and confidentiality obligations.

## Appendix A

**Table A1:** Water applied (mm) in each phenological stage for the five irrigation treatments (T1 = control; T2 = 130 % of T1; T3 = 75 % of T1; T4 = 50 % of T1; T5 = 30 % of T1) and effective rainfall recorded during the 2022/2022 and 2022/2023 seasons.

Phenological Stage	T1	T2	T3	T4	T5	Effective Rainfall
**Season 2022/2022**						
Pre-emergence	20.0	26.0	15.0	10.0	6.0	0.0
Emergence	35.2	45.8	26.4	17.6	10.6	0.0
Vegetative development	28.6	37.2	21.5	14.3	8.6	0.0
Tuber initiation	19.2	25.0	14.4	9.6	5.8	26.1
Flowering initiation	45.8	59.5	34.3	22.9	13.7	26.1
Flowering	49.3	64.1	37.0	24.6	14.8	0.0
Fruit formation	53.1	69.0	39.8	26.6	15.9	11.3
Fruitandseed	0.0	0.0	0.0	0.0	0.0	0.0
Senescence	0.0	0.0	0.0	0.0	0.0	0.0
**Total**	**251.2**	**326.6**	**188.4**	**125.6**	**75.4**	**63.5**
**Season 2022/2023**						
Pre-emergence	20.0	26.0	15.0	10.0	6.0	7.1
Emergence	32.0	41.6	24.0	16.0	9.6	0.0
Vegetative development	32.0	41.6	24.0	16.0	9.6	27.2
Tuber initiation	36.3	47.2	27.2	18.1	10.9	0.0
Flowering initiation	47.3	61.5	35.5	23.7	14.2	7.0
Flowering	55.2	71.8	41.4	27.6	16.6	0.0
Fruit formation	46.0	59.8	34.5	23.0	13.8	0.0
Fruit & seed maturation	13.0	16.9	9.8	6.5	3.9	0.0
Senescence	0.0	0.0	0.0	0.0	0.0	0.0
**Total**	**281.8**	**366.4**	**211.4**	**140.9**	**84.6**	**41.3**
